# EGR1 Is Implicated in Right Ventricular Cardiac Remodeling Associated with Pulmonary Hypertension

**DOI:** 10.3390/biology11050677

**Published:** 2022-04-28

**Authors:** Maria Laggner, Felicitas Oberndorfer, Bahar Golabi, Jonas Bauer, Andreas Zuckermann, Philipp Hacker, Irene Lang, Nika Skoro-Sajer, Christian Gerges, Shahrokh Taghavi, Peter Jaksch, Michael Mildner, Hendrik Jan Ankersmit, Bernhard Moser

**Affiliations:** 1 Department of Thoracic Surgery, Medical University of Vienna, 1090 Vienna, Austria; laggner.maria@gmail.com (M.L.); jonas.bauer@meduniwien.ac.at (J.B.); shahrokh.taghavi@meduniwien.ac.at (S.T.); peter.jaksch@meduniwien.ac.at (P.J.); hendrik.ankersmit@meduniwien.ac.at (H.J.A.); 2 Applied Immunology Laboratory, Medical University of Vienna, 1090 Vienna, Austria; 3 Clinical Institute of Pathology, Medical University of Vienna, 1090 Vienna, Austria; felicitas.oberndorfer@meduniwien.ac.at; 4 Department of Dermatology, Medical University of Vienna, 1090 Vienna, Austria; bahar.golabi@meduniwien.ac.at (B.G.); michael.mildner@meduniwien.ac.at (M.M.); 5 Department of Cardiology, Medical University of Vienna, 1090 Vienna, Austria; andreas.zuckermann@meduniwien.ac.at; 6 Department of Oral and Maxillofacial Surgery, University Hospital St. Poelten, 3100 St. Poelten, Austria; philipp11@gmx.at; 7 Department of Medicine II, Division of Cardiology, Medical University of Vienna, 1090 Vienna, Austria; irene.lang@meduniwien.ac.at (I.L.); nika.skoro-sajer@meduniwien.ac.at (N.S.-S.); christian.gerges@meduniwien.ac.at (C.G.)

**Keywords:** pulmonary hypertension, chronic thromboembolic pulmonary hypertension, idiopathic pulmonary arterial hypertension, right ventricular hypertrophy, reverse right ventricular remodeling, lung transplantation, pulmonary endarterectomy, EGR1

## Abstract

**Simple Summary:**

Pulmonary hypertension (PH) is a condition characterized by increased arterial pressure in the pulmonary vasculature. PH strains the right heart, which compensates for the increased afterload by hypertrophy. This eventually leads to heart failure, which represents the leading cause of death in PH patients. Surgeries normalize pulmonary arterial pressures and cause the regeneration of hypertrophic right hearts. Nonetheless, the events involved in cardiac recovery are largely unknown. We therefore investigated the gene expression profiles of hypertrophic and regenerated right hearts of two different types of PH patients. Intriguingly, the PH subtypes displayed a rather unique gene alteration signature, before as well as after surgery. While genes associated with muscle cell development were upregulated in one group, genes involved in the same molecular process were downregulated in a different PH group following surgery. However, we were able to identify a profibrotic factor, namely early growth response 1, in both PH groups. A role for this molecule in hypertrophic right hearts was further confirmed by immunohistochemistry. In conclusion, our study described the gene expression signatures of failing and recovered right hearts of PH patients. The findings presented here might help to identify attractive therapeutic candidates for PH patients considered inoperable.

**Abstract:**

Background: Pulmonary hypertension (PH) is a vasoconstrictive disease characterized by elevated mean pulmonary arterial pressure (mPAP) at rest. Idiopathic pulmonary arterial hypertension (iPAH) and chronic thromboembolic pulmonary hypertension (CTEPH) represent two distinct subtypes of PH. Persisting PH leads to right ventricular (RV) hypertrophy, heart failure, and death. RV performance predicts survival and surgical interventions re-establishing physiological mPAP reverse cardiac remodeling. Nonetheless, a considerable number of PH patients are deemed inoperable. The underlying mechanism(s) governing cardiac regeneration, however, remain largely elusive. Methods: In a longitudinal approach, we profiled the transcriptional landscapes of hypertrophic RVs and recovered hearts 3 months after surgery of iPAH and CTEPH patients. Results: Genes associated with cellular responses to inflammatory stimuli and metal ions were downregulated, and cardiac muscle tissue development was induced in iPAH after recovery. In CTEPH patients, genes related to muscle cell development were decreased, and genes governing cardiac conduction were upregulated in RVs following regeneration. Intriguingly, early growth response 1 (*EGR1*), a profibrotic regulator, was identified as a major transcription factor of hypertrophic RVs in iPAH and CTEPH. A histological assessment confirmed our biocomputational results, and suggested a pivotal role for EGR1 in RV vasculopathy. Conclusion: Our findings improved our understanding of the molecular events driving reverse cardiac remodeling following surgery. EGR1 might represent a promising candidate for targeted therapy of PH patients not eligible for surgical treatment.

## 1. Introduction

Pulmonary hypertension (PH) is a vasoconstrictive, fibrotic disease characterized by vascular remodeling. Reduced vessel diameter leads to increased resistance and pressure in the pulmonary vasculature [1,2]. In 2015, the consensus guidelines of the European Society of Cardiology (ESC) and the European Respiratory Society (ERS) defined PH as an increased mean pulmonary arterial pressure (mPAP) ≥25 mmHg at rest [3,4]. Currently, the definition of PH is being updated to an mPAP threshold >20 mmHg [5,6]. Various underlying causes that can lead to PH have been identified, such as certain cardiac and pulmonary conditions, systemic diseases, infections, hereditary diseases, and cancer [7]. The World Health Organization (WHO) recognizes five distinct classes of PH, including pulmonary arterial hypertension (PAH, group 1) and chronic thromboembolic PH (CTEPH, group 4) [8].

Based on disease etiology, PAH can be further divided into different subgroups. Pleiotropic conditions can lead to PAH, such as mutations of specific loci, certain drug treatments, congenital heart disease, liver disease, human immunodeficiency virus infection, or autoimmune diseases. However, the exact underlying cause remains unknown in the majority of PAH cases, which are collectively referred to as idiopathic PAH (iPAH). The incidence of PAH was determined as six cases per million [9]. Even with clinical management, five-year survival rates of patients suffering from PAH do not exceed 60% [10]. PAH is characterized by a distinct sequence of pathologic events. PAH manifests with lesions of small pulmonary arteries (<500 µm in diameter) [11], where hypertrophic, proliferative, and fibrotic alterations occur. In addition, inflammatory infiltrates and thrombofibrotic depositions contribute to PAH pathology. The obstructive remodeling of pulmonary vasculature, together with vasoconstriction, inflammation, and thrombosis, lead to increased pulmonary vascular resistance (PVR) and PAP. The elevated pressure is propagated to the heart, where it leads to right ventricular pressure overload. The right ventricle (RV) compensates for chronic hemodynamic overload by hypertrophy and fibrotic remodeling to preserve physiological cardiac output. Proinflammatory milieus, oxidative stress, and humoral responses are contributing second hits for RV overload. RV insufficiency and, eventually, heart failure are the leading causes of death, and importantly, patient prognosis is associated with RV performance [12]. Pharmacological strategies usually involve administration of prostacyclin analogs and inhibitors of the endothelin-1 receptor or phosphodiesterase-5. Isolated, bilateral lung transplantation (LuTX) re-establishes normal PVR and PAP and, hence, reduces RV overload. Though most fibrotic conversions are considered irreversible [13], geometric remodeling was assessed by echocardiography and cardiac morphology reverted to the predisease state three months after LuTX [14,15].

Patients suffering from CTEPH present with dyspnea, exercise intolerance, chest pain, recurrent syncope, and edemas of the lower extremities [16,17]. In Germany, the incidence of CTEPH was reported to be 5.7 cases per million [18]. A CTEPH incidence of up to 9.1% was reported after pulmonary embolism (PE) [19], although 25% of cases of CTEPH had no previous history of PE [20]. CTEPH results from unresolved, vascular obstruction caused by depositions of fibrotic thrombi in the pulmonary arteries. These depositions, in turn, lead to increased resistance and arterial pressure. In most CTEPH patients, the proximal pulmonary arteries are predominantly affected by obstructive alterations. PAH and CTEPH share a similar arteriopathy, which includes fibrosis, blood vessel thickening, and hyperproliferation [21]. The exact underlying etiology for CTEPH remains widely unclear. Dysfunctions in the blood coagulation cascade [22] and impaired angiogenesis [23] have been identified to predispose to CTEPH. Furthermore, inflammatory processes promote CTEPH [24] and various proinflammatory cytokines were elevated in CTEPH patients [21]. Similar to PAH, advanced CTEPH causes myocardial remodeling, including RV dilation and hypertrophy. RV failure is the leading cause of death for patients suffering from CTEPH. Pulmonary endarterectomy (PEA) and balloon pulmonary angioplasty (BPA) represent the gold standard surgical techniques, and up to 90% of surgically treated patients are considered cured. Improved cardiac outcomes have been observed after successful PEA [25,26] and BPA [27]. However, up to 32% of all CTEPH cases are judged inoperable. If left untreated, 76% of patients succumb to CTEPH and sequelae within three years after diagnosis [28].

Early growth response 1 (EGR1) is a key regulator of tendon, cartilage, bone, and adipose tissue formation, homeostasis, and healing. Hence, EGR1 target genes encode for components of the extracellular matrix (ECM). Furthermore, EGR1 was implicated in regulation of fibrotic processes observed in systemic sclerosis, rheumatoid arthritis, and type 2 diabetes mellitus (reviewed in [29]). In addition to these functions, EGR1 governed vascular remodeling in experimental PAH [30], and high EGR1 levels were detected in advanced vascular lesions of patients suffering from advanced-stage congenital heart disease-associated PAH [31]. Though EGR1 has already been implicated in pathological remodeling of the pulmonary vasculature, a potential role of EGR1 in PH-induced RV remodeling has not been investigated to date.

**Figure 1:**
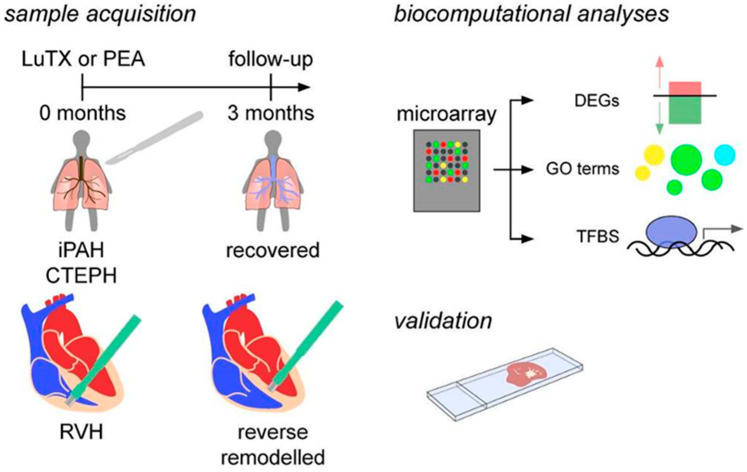
Study design. Cardiac biopsies were acquired intraoperatively and 3 months after surgery. Transcriptional signatures were investigated using a microarray and further biocomputational analyses. Lastly, results were validated using immunocytochemistry. CTEPH, chronic thromboembolic pulmonary hypertension; DEGs, differentially expressed genes; GO, gene ontology; iPAH, idiopathic pulmonary arterial hypertension; LuTX, lung transplantation; PEA, pulmonary endarterectomy; RVH, right ventricular hypertrophy; TFBS, transcription factor binding site.

The pathological alterations associated with PH contribute to increased PVR, which is propagated to the heart, where it manifests as pressure overload to the right ventricle [32]. Sustained RV pressure results in RV dilation and compromised contractile forces. Heart fibrosis predominantly accounts for decreased myocardial function following RV remodeling. As RV performance is associated with patient prognosis [12], and heart failure represents the leading cause of death in PH patients [33], deepening our understanding of RV remodeling is of great importance to improve clinical outcome. Therefore, we compared the diseased myocardium with regenerated RV after (semi)elective surgery to identify key events orchestrating myocardial recovery (Figure 1). These insights might help to unravel hitherto unknown therapeutic targets to promote reversal of pathological cardiac alterations.

## 2. Materials and Methods

### 2.1. Study Population

This study was designed as a prospective, longitudinal, cross-sectional cohort study in 2013. All patients were diagnosed as recommended by applicable guidelines [4,34]. PEA and LuTX surgery were carried out at a single European thoracic surgery center (Department of Thoracic Surgery, Medical University of Vienna, Vienna, Austria). The diagnosis of CTEPH and indication for PEA surgery were performed by teams of specialists. All patients with CTEPH were classified according to the intraoperative classification system [35]. Preoperative assessments included computed tomography of the chest, right heart catheterization with hemodynamic measurements and conventional pulmonary angiography, transthoracic echocardiography, and pulmonary function studies in each patient. Evaluation and listing of iPAH patients for lung transplantation was performed by a multidisciplinary lung transplant team of the Medical University of Vienna according to applicable guidelines [4,34].

### 2.2. Ethics Statement

Ethical approval was granted by the Institutional Ethics Committee of the Medical University of Vienna, Vienna, Austria (1805/2013). This study was performed in accordance with the Declaration of Helsinki and applicable local regulations. All donors provided written informed consent.

### 2.3. Sample Acquisition

For transcriptional analyses, 4 patients with iPAH undergoing bilateral LuTX on central extracorporeal membrane oxygenation (ECMO) support (Table 1) and 4 patients with CTEPH undergoing PEA on cardiopulmonary bypass in deep hypothermia and circulatory arrest (Table 2) were enrolled (Figure 1). Endomyocardial biopsies (EMB) were obtained intraoperatively and 3 months after surgery. Intraoperative myocardial biopsies (1 mm in diameter) were obtained by transmural biopsy of the right ventricular wall. Three months after surgery, the EMB technique was performed by right heart catheterization based on the AHA/ACCF/ESC scientific statement [36].

For histological assessments, posthumous RVs of PH patients were obtained from the Clinical Institute of Pathology, Medical University of Vienna, Vienna, Austria.

None of the CTEPH patients undergoing PEA required postoperative extracorporeal membrane oxygenation (ECMO) support. There were no perioperative cardiovascular adverse events for patients undergoing PEA, except for temporary supraventricular tachycardia in patient #2. None of the CTEPH patients suffered from valvular heart disease, chronic obstructive pulmonary disease (COPD), or chronic renal insufficiency. None of the CTEPH patients had undergone any sort of cardiothoracic surgery prior to PEA.

### 2.4. Microarray

For transcriptomics analyses, the total RNA of cardiac biopsies was isolated using peqGOLD TriFast (Peqlab, Erlangen, Germany) according to the manufacturer’s instructions. RNA concentrations were determined using a NanoDrop1000 spectrophotometer (Peglab), and RNA quality was assessed using an Agilent 2100 Bioanalyzer (Agilent Technologies, Santa Clara, CA, USA). Transcriptome profiling was carried out by the Genomics Core Facility at the Medical University of Vienna (Vienna, Austria) using a Human Clariom S Array (Thermo Fisher Scientific, Waltham, MA, USA) as recommended by the manufacturer.

### 2.5. Biocomputational Analyses

Transcriptome Analysis Console software (TAC, version 4.0, Thermo Fisher Scientific, Waltham, MA, USA) was used for data analysis, for principal component analysis, to determine differentially expressed genes (DEGs), for volcano plots, and for hierarchical clustering. DEGs were defined as a ≤−2 or ≥2 average fold change with a *p*-value < 0.05. Uncharacterized loci, small nucleolar RNAs, microRNAs, duplicates, and noncoding transcripts were excluded. DEGs were further analyzed with Cytoscape (v3.8.0) [37] using the ClueGO (v2.5.7) plug-in [38]. Biological process, immune system process, molecular function, and KEGG were used to identify the pathways and ontologies. The network specificity and pathway network connectivity (kappa score) were set to medium. The GO terms were visualized using REVIGO software [39].

### 2.6. Identification of Transcription Factor Binding Sites (TFBS)

To determine transcription factors, genes differentially expressed between presurgery and 3-month follow-up were analyzed using the oPOSSUM online tool (v3.0) with the default analysis settings [40]. Only genes displaying a log_2_-transformed expression ≥5.6 before and after surgery were included in downstream analyses.

### 2.7. Immunohistochemistry

Immunohistochemistry was performed according to routine laboratory protocols. The EGR1 was stained using polyclonal rabbit antihuman EGR1 IgG (1 µg/mL, Abcam, Cambridge, UK).

### 2.8. Statistical Analyses

DEGs were compared pre- and postoperatively and statistically evaluated with the TAC software using an empirical Bayes method. The DEGs were defined as a ≤−2 or ≥2 average fold change with a *p*-value < 0.05. The GO term *p*-values were determined using ClueGO, and a Bonferroni step-down correction was used to correct for multiple comparisons [38]. The GO terms displaying *p*-values < 0.05 were considered statistically significant.

## 3. Results

### 3.1. Recovered Hearts of Lung-Transplanted iPAH Patients Displayed Decreased Inflammatory Processes and Increased Cardiac Muscle Development

In this study, four patients diagnosed with iPAH undergoing LuTX were included (three females and one male, mean age of 36 years at time of surgery). The average WHO functional class (WHO-FC) was reduced from 3 before surgery to 1.12 post surgery (Table 1). Additionally, the average preoperative systolic PAP decreased from 155 mmHg to physiological levels (Table 1). These data showed that surgical treatment led to improved hemodynamics and clinical status of our patient cohort.

**Figure 2:**
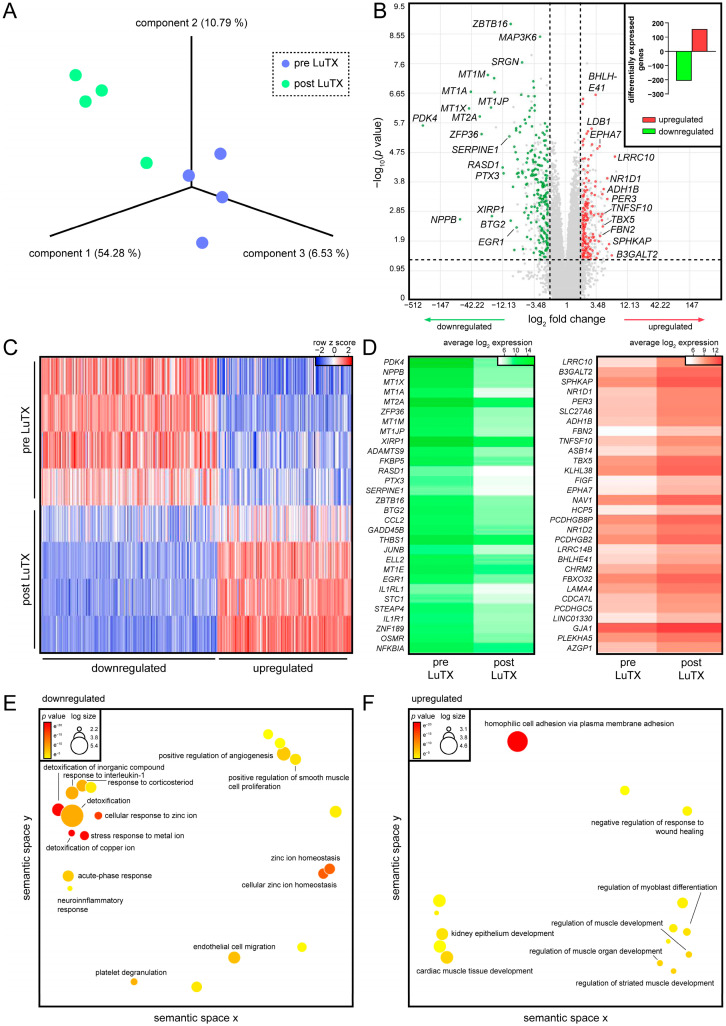
Gene expression signatures of hypertrophic myocardium of iPAH patients before LuTX and reverse remodeled hearts 3 months after surgery. (**A**) PCA of samples before PEA and 3 months after PEA. Each dot represents one donor; colors indicate time points. (**B**) Volcano plot depicting DEGs as a function of fold change and statistical significance. Each dot represents one gene. Red and green indicate up- and downregulated genes, respectively. Insert shows absolute numbers of DEGs when comparing before surgery and after recovery. Red and green bars indicate up- and downregulated genes, respectively. (**C**) Heatmap of DEGs. Each row represents one donor at a certain time point; each line indicates one gene. Red and blue indicate up- and downregulation, respectively. (**D**) Heatmaps of down- (green) and upregulated genes (red) are shown. Colors indicate absolute, average log_2_-transformed expression values. Gene ontologies associated with (**E**) down- and (**F**) upregulated DEGs. Each circle represents one ontology. Colors indicate *p*-values.

First, the global disease signature of dysfunctional RVs of patients suffering from iPAH was profiled. Then, the molecular profile to RVs after reverse remodeling of the same patients 3 months after LuTX was determined and compared to the preoperative status. A principal component analysis (PCA) revealed a distinct subclustering of all samples according to pre- and postsurgery, indicating that LuTX induced remarkable transcriptional changes in RVs (Figure 2A). Genes differentially expressed before and after surgery were determined, and a total of 157 genes were found to be upregulated, while 207 were downregulated (Figure 2B, Supplemental File S1). An analysis of individual donors uncovered a highly similar regulation of DEGs throughout all patients, indicating negligible individual differences (Figure 2C). Pyruvate dehydrogenase kinase 4 (*PDK4*, *p*-value = 2 × 10^−6^), natriuretic peptide B (*NPPB*, *p*-value = 0.0026), several genes encoding for metallothioneins, serpin family E member 1 (*SERPINE1*, *p*-value = 5 × 10^−6^), JunB proto-oncogene (*JUNB*, *p*-value = 2 × 10^−5^), *EGR1* (*p* value = 0.0046), and interleukin-1 receptor-like 1 (*IL1RL1*, *p*-value = 0.0001) were strongly expressed in hypertrophic ventricles, but displayed low expression in recovered hearts (Figure 2D). Leucine rich repeat containing 10 (*LRRC10*, *p*-value = 2 × 10^−5^), beta-1,3-galactosyltransferase 2 (*B3GALT2*, *p*-value = 0.037), nuclear receptor subfamily 1 group D member 1 (*NR1D1*, *p*-value = 0.0001), fibrillin 2 (*FBN2*, *p*-value = 0.0044), and tumor necrosis factor superfamily 10 (*TNFSF10*, *p*-value = 0.0017) were among the top upregulated genes in reverse remodeled RVs compared to presurgery hearts (Figure 2D). To determine the biological processes associated with DEGs, we performed a gene ontology (GO) enrichment analysis. As several metallothionein genes were downregulated, cellular responses to metal ions were found (Figure 2E). In addition, inflammatory processes, such as responses to interleukin 1 and corticosteroids, were identified in hypertrophic RVs. Upregulated genes were mostly associated with cardiac muscle development (Figure 2F).

### 3.2. PEA of CTEPH Patients Promotes Cardiac Conduction in Regenerated RVs

To study the processes involved in the reverse remodeling of hypertrophic RVs, the hearts of four patients diagnosed with CTEPH undergoing PEA were analyzed (two females and two males, average age of 58 years at time of PEA). The mPAP declined from an average of 34 before surgery to 25 after surgery (Table 2). Moreover, the PVR decreased from 6.7 to 2.5 after surgery. The average cardiac index improved from 2.7 to 3.1 after PEA. While patients displayed a WHO-FC of 3 before surgery, the average functional class was reduced to 1.3 following surgery. These data demonstrated a functional and hemodynamic improvement of CTEPH patients after PEA.

**Figure 3:**
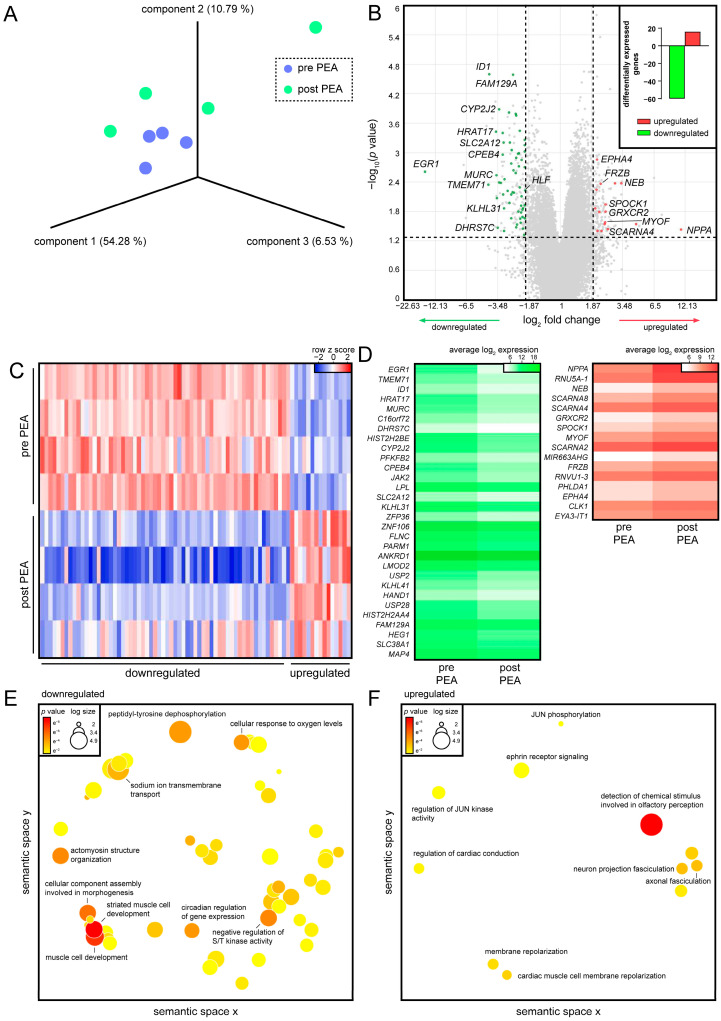
Gene expression profiles of hypertrophic myocardium of CTEPH patients before PEA and reverse remodeled hearts 3 months after surgery. (**A**) PCA of samples before PEA and 3 months after PEA. Each dot represents one donor; colors indicate time points. (**B**) Volcano plot depicting DEGs as a function of fold change and statistical significance. Each dot represents one gene. Red and green indicate up- and downregulated genes, respectively. Insert shows absolute numbers of DEGs before surgery and after recovery. Red and green bars indicate up- and downregulated genes, respectively. (**C**) Heatmap of DEGs. Each row represents one donor at a certain time point; each line indicates one gene. Red and blue indicate up- and downregulation, respectively. (**D**) Heatmaps of down- (green) and upregulated genes (red) are shown. Colors indicate absolute, average log_2_-transformed expression values. Gene ontologies associated with (**E**) down- and (**F**) upregulated DEGs. Each circle represents one ontology; colors indicate *p*-values.

Next, we analyzed the transcriptional changes occurring in the myocardia of CTEPH patients before PEA and after recovery. PCA revealed high interdonor transcriptional similarity before surgery, while patients showed a higher diversity in response to PEA (Figure 3A). In total, 16 genes were found upregulated after recovery, while 60 genes were highly expressed before surgery but downregulated after PEA (Figure 3B, Supplemental File S2). Individual assessment of DEGs uncovered that interdonor differences mostly occurred in the downregulated genes (Figure 3C). *EGR1* (*p*-value = 0.0024), transmembrane protein 71 (*TMEM71*, *p*-value = 0.0045), muscle-restricted coiled-coil protein (*MURC*, *p*-value = 0.0029), cytoplasmic polyadenylation element binding protein 4 (*CPEB4*, *p*-value = 0.0011), janus kinase 2 (*JAK2*, *p*-value = 0.0004), and kelch-like family member 31 (*KLHL31*, *p*-value = 0.0138) were detected among the downregulated genes (Figure 3D). Natriuretic peptide A (*NPPA*, *p*-value = 0.037), nebulin (*NEB*, *p*-value = 0.0042), testican 1 (*SPOCK1*, *p*-value = 0.0113), myoferlin (*MYOF*, *p*-value = 0.0265), and frizzled-related protein (*FRZB*, *p*-value = 0.0043) were found to be upregulated after recovery (Figure 3D). In contrast to the results obtained in iPAH patients, the molecular function muscle cell development was associated with downregulated genes in CTEPH after PEA (Figure 3E). Interestingly, upregulated genes were involved in cardiac conduction and cardiac muscle cell membrane repolarization (Figure 3F). These data indicated a PH class-specific transcriptional regulation that contributed to improved cardiac performance after surgery.

### 3.3. EGR1 Is Implicated in Reverse Remodeling of RVH

**Figure 4:**
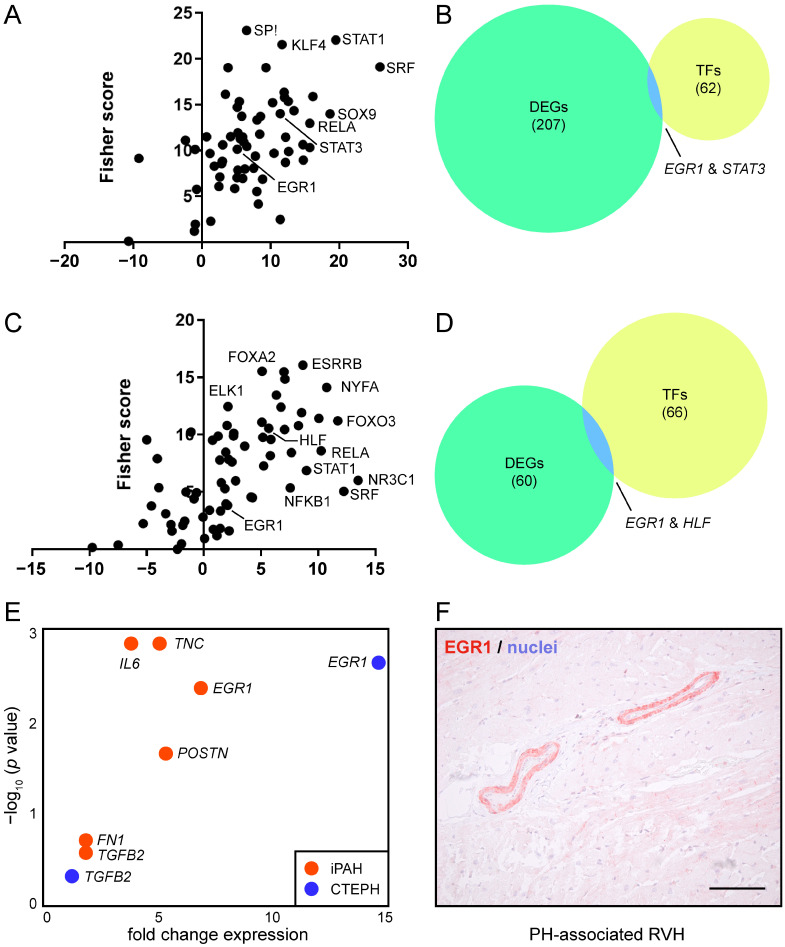
Identification of TFs governing postoperative reverse cardiac remodeling. (**A**) Genes highly expressed preoperatively and downregulated 3 months after surgery were screened for TFBS. Factors identified in (**A**) iPAH and (**C**) CTEPH are depicted as a function of z- and Fisher scores. An area-proportional Venn diagram of overlapping DEGs and TFs identified by TFBS analysis in (**B**) iPAH and (**D**) CTEPH patients. Numbers indicate absolute number of genes. (**E**) Average expression values of EGR1 downstream genes of iPAH and CTEPH patients. Fold-change expression was determined by comparing pre- and postsurgery values. (**F**) Histocytochemistry of EGR1 in the hypertrophic right ventricle of a PH patient. One representative micrograph of *n* = 3 donors is shown. Scale bar = 100 µm.

Next, we sought to determine the transcription factors (TFs) that potentially acted as master transcriptional regulators of RV reverse remodeling. To this end, we used a web-based platform to identify the TF binding sites (TFBS) present in the promoter regions of DEGs. As we aimed to investigate factors highly prevalent in the diseased myocardium, we focused our analysis on downregulated genes. We were able to identify 62 potential TFs in the downregulated genes of iPAH patients, such as Krüppel-like factor 4 (KLF4), signal transducer and activator of transcription 1 and 2 (STAT1 and STAT3), SRY-box transcription factor 9 (SOX9), and EGR1 (Figure 4A, Supplemental File S3). We then screened the list of TFs for the presence of downregulated genes, and detected EGR1 and STAT3 in both datasets (Figure 4B).

We performed the same analysis for CTEPH, and found forkhead box A2 (FOXA2), ETS transcription factor ELK1 (ELK1), STAT1, and EGR1 among the identified 66 TFs (Figure 4C, Supplemental File S4). When comparing the lists of TF with DEGs, we found the common factors EGR1 and hepatic leukemia factor (HLF) (Figure 4D).

As the analyses of the iPAH and CTEPH data sets both identified EGR1 as a transcriptional regulator of reverse remodeling, we focused our further analyses on EGR1. Several EGR1 downstream genes, such as periostin (*POSTN*), tenascin C (*TNC*), fibronectin (*FN1*), and transforming growth factor beta 2 (*TGFB2*), were highly expressed in the diseased hearts of iPAH and CTEPH patients (Figure 4E). Lastly, immunohistochemistry of EGR1 in the hypertrophic RV of PH patients corroborated our bioinformatics results (Figure 4F). Intriguingly, EGR1 tissue expression was predominantly detected in the tunica media of blood vessels, while tunica intima and tunica adventitia were devoid of EGR1 staining. Together, these data suggested a role of EGR1 in RV remodeling.

## 4. Discussion

Numerous studies have investigated the underlying and perpetuating causes of PH, and over the past decades, our understanding of the pathomechanistic events governing disease onset and progression has improved profoundly. Vascular remodeling [41], endothelial-to-mesenchymal transition [42], sRAGE signaling [43], and EGFR signaling [44,45,46] are among the cellular and molecular processes known to be implicated in PH disease onset and/or progression. However, the majority of studies investigating pulmonary hypertension focused on the pulmonary vasculature [47,48,49]. While pulmonary processes represent a central aspect of PH, the relevance of cardiac performance is often neglected in PH basic research and clinical management. Most therapeutic strategies for PAH directly aim at lowering the mPAP, though reduced pulmonary pressure does not necessarily lead to improved patient conditions unless RV hemodynamics and RV functions recover [50]. Elucidating the molecular mechanisms governing PH-associated RVH and subsequent reverse remodeling is therefore of utmost interest, and promoting reverse RV remodeling might represent a major advancement in the therapy of PH. In our current study, we therefore investigated the transcriptional signatures of diseased and regenerated right ventricles of PH patients before surgery and after recovery. By longitudinal comparison, we were able to detect a vast array of DEGs, including several genes associated with muscle development. When screening for transcriptional regulators, we identified EGR1 as an instigator of right ventricular remodeling in iPAH and CTEPH patients. Histological investigations suggested a potential role for EGR1 in PH-related vasculopathy of the right ventricle.

The results reported in our study allowed a detailed view of the transcriptional landscape of PH-associated hypertrophic RVs, and provided insights into the transcriptional changes occurring after surgery and after RV performance recovery. We identified several differentially regulated factors that have already been implicated in various cardiopathological events, such as PDK4 [51,52,53], metallothioneins [54,55,56], XIRP [57], SERPINE1 [58], LRRC10 [59,60,61], NR1D1 [62], fibrillin-1 [63], MURC [64,65], CPEB4 [66], JAK2 [67], members of the KLHL [68], NEB [69], and MYOF [70]. As these genes displayed a distinct regulation in iPAH and CTEPH patients, PH subgroup-specific mechanisms seemed to orchestrate reverse remodeling of hypertrophic RVs. Our data suggested several potential factors and pathways that conceivably contributed, in a synergistic and/or in a parallel manner, to RV recovery. Future mechanistic and functional studies are warranted to determine the role of the identified factors in heart regeneration.

No single event driving RV maladaptation has been found so far. Rather, multifactorial concomitants have been identified to play crucial roles in the conversion of compensatory RV hypertrophy to RV dilation and failure. Neurohormones are considered key players in PH-related RV dysfunction. Angiotensin II, angiotensin-converting enzymes (ACEs), prostaglandins, and natriuretic peptides are reportedly involved in RV remodeling [33]. While angiotensin (*AGT*) was not differentially expressed in hearts of iPAH and CTEPH patients, we observed a trend toward decreased angiotensin I converting enzyme 2 (*ACE2*) in CTEPH and increased angiotensin I converting enzyme (*ACE*) in iPAH after surgery. ACEs displayed a highly PH group-specific regulation, and investigating larger patient cohorts will be required to determine the specific roles of ACEs in different classes of PH. Among all prostaglandin genes, we detected diminished prostaglandin D2 synthase (*PTGDS*) expression in both iPAH and CTEPH hearts following surgery, although with a less pronounced decrease in CTEPH. These data were in line with a previous study that reported the overexpression of prostaglandin D synthases in hypertrophic hearts [71], further underlining the importance of prostaglandin-synthesizing enzymes in cardiac hypertrophy.

Interestingly, we observed a remarkable upregulation of *NPPA* and, to a lesser extent, *NPPB* after PEA. In contrast to these findings, *NPPB* was strongly downregulated in hearts of iPAH patients after LuTX, and *NPPA* also displayed a tendency toward decrease. These data indicated an intricate regulation of natriuretic peptides in PH-associated right heart pathology. Mice lacking *Nppa* developed cardiomyocyte hypertrophy [72,73], hence the decreased expression of *NPPA* we detected in diseased hearts of CTEPH patients might represent a potential mechanism for hypertrophy. Since *Nppb* is considered a marker for cardiac hypertrophy [74,75], the high *NPPB* expression detected in hypertrophic hearts of iPAH patients might serve as an indicator of cardiac hypertrophy in PH patients as well.

Several pathways guiding heart development have also been implicated in maladaptive cardiac hypertrophy. As such, WNT signaling is crucial for cardiogenesis, but also mediated adult heart remodeling [76]. However, FRZB inhibited Wnt-mediated cell proliferation in cardiac cushions [77]. Our analyses unveiled low *FRZB* expression before PEA, and it is tempting to speculate that a lack of *FRZB* might lead to elevated WNT signaling and cardiac remodeling. However, further studies will be required to fully elucidate the exact role of the WNT–FRZB axis in cardiac hypertrophy resulting from CTEPH.

In our study, we identified EGR1 as a major instigator of RVH in both iPAH and CTEPH patients. EGR1 was strongly expressed in diseased, hypertrophic hearts, and was remarkably downregulated following surgical intervention and reverse RV remodeling. Furthermore, EGR1 was found to act as a transcriptional regulator of a number of DEGs. Finally, we were able to confirm our findings in a histological assessment of hypertrophic RVs.

Egr1 was induced in the early response to myocardial infarction (MI) [78], and Egr1 governed expression of several downstream targets implicated in inflammation, thrombosis, and apoptosis following MI [79]. Inhibiting EGR1 by DNAzymes improved cardiac performance after MI [80]. Furthermore, Egr1 played an important role in the early stages of cardiac hypertrophy via transcriptional regulation of T-type calcium channels [81], and Egr1 was reported as an endogenous regulator of pathologic cardiac hypertrophy [82]. These previous reports underpinned a crucial role for EGR1 in cardiac hypertrophy, and the results obtained in the current study further expanded the field of action of EGR1 to cardiac hypertrophy resulting from PH.

Our immunohistochemical staining suggested a role for EGR1 in RVH vasculopathy. It is known that EGR1 is poorly expressed in healthy blood vessel walls [83], and we observed elevated EGR1 in the tunica media of vessels of the RV. Previously, it was shown that endothelial cells rapidly upregulated Egr1 expression following vascular injury [84]. Furthermore, migrating smooth muscle cells were found to be Egr1-positive [85]. Egr1 thus plays an important role in the vascular response to injury and targeting Egr1 by DNAzyme-prevented intimal thickening [86,87]. Similarly, DNAzyme-mediated downregulation of Egr1 attenuated neointimal formation and improved RVH in a rodent experimental model of PAH [30]. In iPAH patients, van der Feen and colleagues reported EGR1 expression in the intima layer, and more than half of the cells of the media layer of pre- and intra-acinar pulmonary vessels [31]. In contrast to this finding, we exclusively observed EGR1 expression in the tunica media of hypertrophic RVs from PH patients. Thus, the molecular processes and contributing cell types that drove remodeling of the pulmonary vasculature and of the RV might be organ-specific. Our immunohistochemical results were further in line with a previous study on the role of Egr1 in coronary allograft vasculopathy [88]. In their work, Okada et al. reported a major role for Egr1 in the parenchymal rejection of cardiac allografts and blocking Egr1 by antisense-DNA-delayed consequences of cardiac rejection. As cardiomyocytes of hypertrophic RVs were largely devoid of EGR1 staining, the potential crosstalk between EGR1-positive blood vessel cells with the adjacent cardiomyocytes and immune cells, either via direct cell–cell interactions or via paracrine signaling, remains to be determined.

Interestingly, AGE/RAGE is an upstream positive regulator of EGR1, and the RAGE axis was locally and systemically upregulated in iPAH and CTEPH [43]. Therefore, a RAGE-dependent induction of EGR1 is conceivable, and investigating the upstream regulatory mechanisms driving EGR1 activation will be the subject of future studies.

Pharmacological targeting of EGR1 remains challenging, as small molecules targeting EGR1 have not been identified to date [89]. However, the growing body of previous reports together with our findings strongly encourage the development of EGR1 inhibitors. Whether modulating EGR1 signaling prevents RV deterioration in PH patients merits future investigations.

As a result of different etiologies, iPAH displayed an earlier disease onset compared to CTEPH. Hence, it is tempting to speculate that younger iPAH patients might have a better recovery after surgery. Future studies are warranted to determine whether improvement of postoperative hemodynamic parameters is related to patient age.

### Limitations

In spite of our best efforts, we must recognize some limitations of our study. We analyzed a limited number of patient samples, and investigating larger patient cohorts might reveal further differences and alterations in reverse RV remodeling that were not captured here. For example, stratification of hemodynamic and transcriptional data according to patient age might unveil valuable new insights. Due to the study design, only patients undergoing surgery were included. PH patients deemed inoperable but receiving medical therapy or rehabilitation might display a distinct disease signature compared to operable PH patients in the presurgery state. Uncovering the differences between operable and inoperable PH patients remains to be determined. Due to ethical and medical reasons, tissue samples were acquired from different anatomical locations of the heart. Though we were able to confirm our bioinformatics data in the RVs of PH patients, EGR1 staining of recovered RVs will be necessary to further corroborate our findings. Furthermore, different medical therapies were prescribed for iPAH and CTEPH patients, before surgery as well as after surgery. Elucidating the potential effect of different medications of RV remodeling will be the subject of future investigations.

## 5. Conclusions

Our findings hold great promise for identifying disease biomarkers and for discovering diagnostic options. Promoting reverse ventricular remodeling using targeted therapy might help reducing postoperative mortality. The results presented here might further allow identification of molecular candidates to treat PH patients not eligible for surgical treatment.

## Figures and Tables

**Table 1:** Basic demographic and hemodynamic data of iPAH patients.

Patient ID	1	2	3	4
Gender	F	F	F	M
Age at BLTX (years)	27	38	39	40
Type of LuTX	BLTX size reduced: resection of ML and lingula	Lobar TX: RLL and LUL	Lobar TX: RLL and LUL	Size-reduced BLTX: ML resection
Preop WHO-FC	3	3	3	3
Postop WHO-FC	1	1	0.5	2
Preop 6-MWD (m)	300	160	-	-
Preop PH-specific treatments	Double-therapy	Triple-therapy	Double-therapy	Double-therapy
Postop cardiological medication (s)	Bisoprolol	Bisoprolol	Ramipril	Nitrendipin, ivabradin, molsidomin
**Pre- and Postoperative Hemodynamics**				
Preop PAPsys (mmHg)	103	180	168	168
Postop PAPsys (mmHg)	No TR signal *	No TR signal *	No TR signal *	46

* No tricuspid regurgitation (TR) signal measurable, indicative of a healthy RV. BLTX, bilateral lung transplantation; F, female; LUL, left upper lobe; LuTX, lung transplantation; M, male; ML, middle lobe; PAPsys, systolic pulmonary arterial pressure; postop, postoperative; preop, preoperative; RLL, right lower lobe; TX, transplantation; TR, tricuspid regurgitation; WHO-FC, World Health Organization functional class; 6-MWD, six-minute walk distance.

**Table 2:** Basic demographic and hemodynamic data of CTEPH patients.

Patient ID	5	6	7	8
Gender	M	F	F	M
Age at PEA (years)	54	61	66	50
PA:AA ratio	1.06	1.33	1.46	0.97
History of VTE	PE 9 months prior to PEA	PE 3 months prior to PEA	DVT and PE 3 years prior to PEA	DVT and PE 1 year prior to PEA
Preop PH-specific medications	None	None	LTOT	Riociguat
Postop PH-specific medications	None	None	None	None
CAD or stenosis	Yes	No	LAD stenosis type B1: 70–90% *	No
Concomitant surgery	CABG 2-vessel surgery	No	No	No
Comorbidities	Chronic bronchitis	History of ileus, hysterectomy	Heterozygote prothrombin SNP G20210A, psoriasis arthritis	Arterial hypertension, asthma
UCSD classification of surgical specimens	2	3	3	3
Preop WHO-FC	3	3	3	3
Postop WHO-FC	1	2	1	1
**Pre- and Postoperative Hemodynamics**				
Preop PAP (s/d/m) (mmHg)	75/24/40	64/24/40	PAPm 26	55/22/36
Postop PAP (s/d/m) (mmHg)	22/11/16	62/23/33	32/13/20	38/17/25
Preop PVR (WU)	4.19	9.61	6.95	5.95
Postop PVR (WU)	1.24	4.31	1.76	2.82
Preop CI (L/min/m^2^)	2.9	3.1	2.8	2.1
Postop CI (L/min/m^2^)	3.1	2.2	3.8	3.2

* LAD stenosis resulted from compression of the dilated pulmonary trunk. CABG, coronary artery bypass graft; CAD, coronary artery disease; CI, cardiac index; DVT, deep vein thrombosis; F, female; LAD, left anterior descending artery; LTOT, long-term oxygen therapy; M, male; PA:AA ratio, pulmonary artery diameter to ascending aorta diameter; PAP (s/d/m), pulmonary artery pressure (systolic/diastolic/mean); PE, pulmonary embolism; PEA, pulmonary endarterectomy; PH, pulmonary hypertension; postop, postoperative; preop, preoperative; PVR, pulmonary vascular resistance (in Wood units); SNP, single-nucleotide polymorphism; UCSD, University of California-San Diego; VTE, venous thromboembolism; WHO-FC, World Health Organization functional class.

## Data Availability

Raw data are available upon request.

## Supplementary Materials

The following supporting information can be downloaded at: https://www.mdpi.com/article/10.3390/biology11050677/s1, File S1: Differentially expressed genes for iPAH, File S2: Differentially expressed genes for CTEPH, File S3: Transcription factor binding sites for iPAH, File S4: Transcription factor binding sites for CTEPH.
